# CzeLex: A megastudy dataset of concreteness, imageability, age of acquisition, valence, and gender ratings for 9601 Czech words

**DOI:** 10.3758/s13428-026-03217-4

**Published:** 2026-11-06

**Authors:** James Brand, Filip Smolík, Jan Chromý, Markéta Ceháková, Mikuláš Preininger

**Affiliations:** 1 Department of Psychology, Edge Hill University, St Helens Rd, Ormskirk, L39 4QP UK; 2 Institute of Psychology, Czech Academy of Sciences, Prague, Czechia; 3 Faculty of Arts, Charles University, Prague, Czechia

**Keywords:** Norms, Word processing, Lexical semantics, Czech, Megastudy

## Abstract

Understanding how semantics are processed at the lexical level has been a key aim for the cognitive sciences, with large-scale semantic rating studies in different languages playing a crucial role in facilitating the advancement and development of theories of lexical processing, as well as providing valuable methodological resources that can be used in empirical investigations. In this paper, we present the CzeLex database, a megastudy of 9601 Czech words that have been normed along five psycholinguistic dimensions from a large sample of 4405 Czech-speaking participants. We provide individual rating data at the item and participant levels, as well as aggregated data from both mean ratings and latent means derived using cumulative-link mixed modeling, along with a comprehensive analysis comparing aggregation methods. We demonstrate that the dataset has good reliability and that specific dimensions predict lexical processing measures in a lexical decision task, regardless of the aggregation method used. These norms are the largest available subjective rating resource for Czech and further contribute to the growing body of languages with openly available data on these dimensions. We hope that the dataset will be a valuable resource for researchers working on Czech, in addition to opening up new opportunities for cross-linguistic semantic investigations. All data, code, and analyses are available at https://osf.io/tqxfm.

## Introduction

Measuring meaning has long been a goal for interdisciplinary work in the cognitive sciences, linguistics, and psychology, dating back to the early work of Osgood et al. (1957). At the core of this work has been the production of megastudy datasets that provide normative ratings for large sets of items in a given language, which can be used as gold-standard benchmarks for experimental designs or empirical investigations. Semantic dimensions such as concreteness (Brysbaert et al., 2014a) and valence (Warriner et al., 2013) have been a cornerstone of such research, whereby large datasets can be used to quantifiably estimate the semantic representation of an item; for example, knowing that *trombone* is more concrete and positive compared to *tax*, which is more abstract and negative, allows researchers to carefully control and explore thousands of items within a given language.

Much of the work in this area has given a lot of attention to the nature of word meanings, but there has also been a range of theoretical perspectives that have tried to account for the way that semantics are represented, with much of the quantitative work on word meanings being closely tied to these theories. Some approaches assume that word meaning can be represented through relatively stable lexical content, such as semantic features or category structures (e.g., Collins & Quillian, 1969; Rosch & Mervis, 1975). Other accounts place greater emphasis on the role of inference, context, and world knowledge in meaning construction (e.g., Elman, 2009). Distributional approaches, which propose that semantic representations emerge from patterns of linguistic co-occurrence, such that words derive meaning from the contexts in which they appear, have also been influential (e.g., Firth, 1957; Landauer & Dumais, 1997). Finally, embodied perspectives place greater emphasis on the role of perceptual, motor, and affective experiences that ground semantic knowledge (Barsalou, 1999; 2008). Normative datasets that aim to quantify semantic dimensions for the lexicon of a language, such as concreteness and valence, have acted as key resources allowing researchers to test these theories better and, crucially, to address issues related to how we understand semantic representation more broadly (see e.g., Davis & Yee, 2021; Wingfield & Connell, 2022).

The methodological approach taken to collect such normative datasets has been relatively stable, with the design involving a clear definition of the dimension being investigated, a set of items to be normed, and a corresponding scale that can be used by participants to provide their subjective ratings (this is typically an ordinal scale with numeric or text-based choices, but see Hollis & Westbury, 2018, for alternative formats). Given a large enough sample size and a large enough item list, such megastudies can provide coverage with high reliability for a large portion of the human lexicon, meaning that researchers can freely and efficiently address novel research questions in lexical processing (e.g., Kousta et al., 2011), semantic representation (e.g., Wingfield & Connell, 2023), or careful experimental control (e.g., Taylor et al., 2020).

In recent years, there has not only been a shift in focus on which specific semantic dimensions of meaning are being normed, with a wider and diverse range of theoretically motivated datasets being published (e.g., cognition: Corenblum & Pexman, 2025; humor: Engelthaler & Hills, 2018; motivation: Giurgea et al., 2025; social-semantics: Diveica et al., 2023; Preininger et al., 2025; survival: Alonso et al., 2021; Bonin et al., 2024), but there has also been more attention on the types of languages for which these datasets are available. With the majority of studies in the literature focusing on English-speaking samples from either Canada, the US, or the UK, there has been an overreliance on these datasets to study aspects of human cognition, leading to calls for broader linguistic diversity to address questions of generalizability (Berghoff & Bylund, 2025; Blasi et al., 2022; Brysbaert, 2026). The availability of diverse norming datasets across many different languages has been an area for which promising developments have been made, with a growing body of resources openly available across different language families, varieties and modalities. In Table 1, we provide an overview of the available datasets for languages that have, to the best of our knowledge at the time of writing, at least one large-scale^1^ norming study for the five dimensions covered in the current paper. Naturally, having reliable datasets available for researchers to use, leads to more diverse work on language specific phenomena (Smolík & Kříž, 2015), as well as opening up valuable new opportunities for cross-linguistic comparisons (Łuniewska et al., 2016).

**Table 1:** List of languages with at least one human-derived large-scale norming database for either AoA, concreteness, imageability, valence, or gender. The number of stimuli items being rated and the number of participants (ppts) is given, in addition to the citation. Where multiple sources are available for a given language, we refer to the paper with the largest number of items. If a dimension only has a smaller database available in a language, we also include that information, even if it would not be considered large-scale

Language	AoA	Concreteness	Gender	Imageability	Valence
Arabic	Items = 2467, ppts = 803 Alzahrani et al. (2025)	Items = 2467, ppts = 803 Alzahrani et al. (2025)	-	-	-
British sign language	Items = 300, ppts = 33 Vinson et al. (2008)	Items = 234, ppts = 15 Ortega et al. (2025)	-	-	-
Chinese (simplified)	Items = 19,716, ppts = 1765 Xu et al. (2021)	Items = 9877, ppts = 1140 Xu and Li (2020)	-	Items = 1100, ppts = 960 Yao et al. (2017)	Items = 11,310, ppts = 1444 Xu et al. (2022)
Chinese (traditional)	Items = 4376, ppts = 20 Su et al. (2023)	Items = 25,281, ppts = 1080 Chan and Tse (2024)	-	Items = 25,281, ppts = 1080 Chan and Tse (2024)	Items = 25,281, ppts = 1080 Chan and Tse (2024)
Croatian	Items = 6000, ppts = 3630 Peti-Stantić et al. (2021)	Items = 6000, ppts = 3630 Peti-Stantić et al. (2021)	-	Items = 6000, ppts = 3630 Peti-Stantić et al. (2021)	Items = 3022, ppts = 933 Ćoso et al. (2019)
Czech	Items = 299, ppts = 26 Łuniewska et al. (2019)	-	Items = 3999, ppts = 1709 Preininger et al. (2025)	Items = 435, ppts = 122 Smolík (2019)	Items = 3999, ppts = 1709 Preininger et al. (2025)
Dutch	Items = 30,000, ppts = 74 Brysbaert et al. (2014a, b)	Items = 30,000, ppts = 75 Brysbaert et al. (2014a, b)	Items = 24,037, ppts = 80 Vankrunkelsven et al. (2024)	Items = 4600, ppts = NA van Loon-Vervoom (1985)	Items = 24,000, ppts = 94 Speed and Brysbaert (2024)
English	Items = 30,121, ppts = 1960 Kuperman et al. (2012)	Items = 39,954, ppts = 4000 Brysbaert et al. (2014a, 2014b)	Items = 5553, ppts = 829 Scott et al. (2019)	Items = 5553, ppts = 829 Scott et al. (2019)	Items = 13,915, ppts = 1827 Warriner et al. (2013)
Estonian	-	Items = 35,979, ppts = 2278 Proos and Aigro (2024)	-	-	-
Finnish	Items = 299, ppts = 24 Łuniewska et al. (2019)	-	-	-	Items = 420, ppts = 996 Söderholm et al. (2013)
French	Items = 1493, ppts = 59 Ferrand et al. (2008)	Items = 1659, ppts = 482 Bonin et al. (2018)	-	Items = 1493, ppts = 30 Bonin et al. (2011)	Items = 1659, ppts = 482 Bonin et al. (2018)
French sign language	-	Items = 546, ppts = 33 Périn et al. (2024)	-	-	-
German	Items = 3259, ppts = 537 Birchenough et al. (2017)	Items = 1000, ppts = 64 Kanske and Kotz (2010)	-	Items = 2900, ppts = 200 Võ et al. (2009)	Items = 2900, ppts = 200 Võ et al. (2009)
German sign language	Items = 313, ppts = 62 Trettenbrein et al. (2021)	Items = 234, ppts = 18 Ortega et al. (2025)	-	-	-
Indonesian	-	Items = 1490, ppts = 328 Sianipar et al. (2016)	-	-	Items = 1490, ppts = 328 Sianipar et al. (2016)
Italian	Items = 1957, ppts = 507 Montefinese et al. (2019)	Items = 1121, ppts = 400 Montefinese et al. (2014)	-	Items = 1121, ppts = 400 Montefinese et al. (2014)	Items = 1121, ppts = 684 Montefinese et al. (2014)
Persian	Items = 299, ppts = 21 Łuniewska et al. (2019)	-	-	-	Items = 718, ppts = 463 Mahjoubnavaz et al. (2024)
Polish	Items = 4900, ppts = 400 Imbir (2016)	Items = 4900, ppts = 400 Imbir (2016)	-	Items = 4900, ppts = 400 Imbir (2016)	Items = 6000, ppts = 21,878 Wierzba et al. (2022)
Portuguese (European)	Items = 1749, ppts = 685 Cameirão & Vicente (2010)	Items = 3800, ppts = 2357 Soares et al. (2017)	-	Items = 3800, ppts = 2357 Soares et al. (2017)	Items = 1034, ppts = 958 Soares et al. (2012)
Serbian	Items = 2100, ppts = 603 Popović Stijačić and Filipović Đurđević (2025)	Items = 2100, ppts = 603 Popović Stijačić and Filipović Đurđević (2025)	-	Items = 2100, ppts = 603 Popović Stijačić and Filipović Đurđević (2025)	Items = 2100, ppts = 603 Popović Stijačić and Filipović Đurđević (2025)
Spanish	Items = 7039, ppts = 2150 Alonso et al. (2015)	Items = 1400, ppts = 826 Guasch et al. (2016)	-	Items = 1400, ppts = 826 Guasch et al. (2016)	Items = 14,031, ppts = 350 Stadthagen-Gonzalez et al. (2017)
Spanish (rioplatense)	-	Items = 1034, ppts = 483 Sarli and Justel (2022)	-	Items = 1034, ppts = 483 Sarli and Justel (2022)	Items = 1034, ppts = 483 Sarli and Justel (2022)
Turkish	Items = 503, ppts = 134 Conca et al. (2024)	Items = 2031, ppts = 1527 Kapucu et al. (2021)	-	Items = 503, ppts = 134 Conca et al. (2024)	Items = 2031, ppts = 1527 Kapucu et al. (2021)

A further development in the literature has been the use of large language models (LLMs) to generate and scale up normative datasets. For example, there now exist LLM-augmented datasets for a range of different psycholinguistic variables (e.g., Conde et al., 2025, 2026; Martínez et al., 2025a, b; Trott, 2024a). Given the efficiency with which these datasets can be created, compared with traditional crowd-sourced datasets from human participants, where financial and time constraints can limit development, the growing popularity of these LLM-derived datasets is understandable (Heyman & Heyman, 2024). Nevertheless, a key consideration of working with LLM-generated data is ensuring that it has been validated against human-generated datasets.

As most of the work emerging from the LLM approach has focused predominantly on English and Spanish – languages where the models have much more training data – having large-scale ‘gold-standard’ reference points derived from human participants in different languages is more pertinent than ever if these approaches are to be validated and evaluated more widely (see Chang & Bergen, 2024; Trott, 2024b). Although researchers have attempted to automatically estimate rating data using word embedding models across multiple languages (e.g., Buechel et al., 2020; Thompson & Lupyan, 2018), it remains unclear when these approaches work most accurately. Thus, human-derived datasets across different languages, which have been shown to be validated and have sufficiently large samples of participants and items, remain a vital and irreplaceable resource for researchers to better test theories of word meaning and address issues related to theoretical accounts of semantic representation.

## The CzeLex norms

In this paper, we introduce the CzeLex norming database, a multidimensional set of psycholinguistic estimates for 9601 Czech words. Each word has human-derived ratings from a total sample of 4405 participants along five distinct dimensions of semantic representation: concreteness, imageability, age of acquisition, valence, and gender, representing the largest available resource of psycholinguistic norms for Czech. We will first outline some properties of Czech that highlight why it is a language of interest from a psycholinguistic perspective, followed by an introduction to the dimensions of interest from a theoretical standpoint and the motivation for their inclusion in the database. Next, we justify the need for different types of summative data for norming studies, emphasizing the need for statistics beyond the mean, especially for ordinal-based Likert scales. Finally, we outline the key aims for the present study and outline the way the norms will be validated and tested for reliability, ensuring that they can act as a ‘gold-standard’ reference point for the Czech language.

## Linguistic properties of Czech

Czech is a Slavic language with properties that make it a valuable source for studying different aspects of language processing, acquisition, and representation, not only from a general perspective but also when examining comparative research questions. It has highly inflectional morphology, with most nouns, verbs, adjectives, numerals, and pronouns appearing in different forms depending on the syntactic structures in which they are used. Although the focus of the current paper is not on whether the semantic properties of words differ across individual inflectional forms (for example, it would not be expected that *okno* [window] would exhibit any large shifts in semantic representations when presented in different inflectional variants, e.g. *okno, okna, oknu, okně, oknem, oken, oknům, oknech, okny),* there is evidence that semantic characteristics tend to be associated with formal features such as length, and it is possible that such association also exists with inflectional properties such as number of distinct inflected forms (Reilly & Kean, 2007) but these possibilities cannot be properly addressed in languages with limited or absent inflectional systems. Furthermore, Czech has a highly transparent orthography, where the correspondence between graphemes and phonemes is predictable. Semantic variables such as imageability have been shown to affect reading in languages with opaque orthography (Bakhtiar & Weekes, 2015; Ellis & Lambon Ralph, 2000), but the pattern of effects could differ in a transparent system. Taken together, these language-specific properties could let us use the data in novel ways to better understand how structural features may interact with semantic representations during tasks such as reading.

## Dimensions being normed

Below, we outline the theoretical and empirical background for each of the five dimensions for which there is data within the CzeLex norms. We focused on these dimensions to ensure there is a multidimensional dataset available of distinct semantic norms for Czech, as was the case for other languages (e.g., Scott et al., 2019). Whilst there are a number of dimensions being presented in this paper, our main motivation for choosing this selection of dimensions was to provide researchers with a range of dimensions that have received considerable attention in the existing literature; i.e., AoA, imageability, concreteness, and valence have all been studied in detail in other languages, but we also include another dimension – gender – which has received substantially less attention, as highlighted by Table 1. We acknowledge that this is not an exhaustive list and there are many other theoretically motivated dimensions of semantic representation (see Binder et al., 2016) that could be further investigated to better empirically understand semantic representations.

### Age of acquisition (AoA)

This dimension captures the age at which a participant subjectively assesses an item to have been acquired. Although there are a number of different methodological ways to measure this (e.g., test-based: Brysbaert & Biemiller, 2017; corpus-based: Grimm et al., 2019; Smolík & Filip, 2022), it is typically estimated by asking adults to provide an estimate of the age at which they first understood the word (Kuperman et al., 2012), with values ranging from very early (e.g., *mommy* to very late (e.g., *taxonomy*). It has been argued that words learned earlier in life are easier to process, even when controlling for lexical variables such as frequency and length (Monaghan & Ellis, 2010; Zevin & Seidenberg, 2002), as well as predicting retention in cognitive impairment (Brysbaert & Ellis, 2016).

### Concreteness

This dimension captures an aspect of sensorimotor experience, where participants are typically asked to judge the extent to which the item is either abstract (not experienced through any sensory modality or motor responses, e.g., *hydrogen*) or concrete (greatly experienced through one or more sensory modalities or motor responses, e.g., *lettuce*). There is an acknowledgment that a unidimensional approach to sensorimotor experience through an abstract/concrete continuum leads to a larger bias towards visual and haptic experiences and underestimates other modalities (Brysbaert et al., 2014b; Connell & Lynott, 2012). This has led to a range of different approaches to capture the underlying construct of sensorimotor experience, such as through multidimensional ratings (Lynott et al., 2020) or more specialized definitions (e.g., body–object interaction: Pexman et al., 2019; sensory experience ratings: Juhasz & Yap, 2013). Nevertheless, the theoretical importance of concreteness has allowed for a deeper understanding of the way certain concepts are processed, acquired, and represented, with a focus on embodied or grounded accounts of conceptual representation (Barsalou, 2020; Borghi et al., 2019).

### Gender

This dimension captures the semantic representation of gender for concepts, whereby items are typically rated along a bipolar scale anchored by feminine (e.g., *womb*) and masculine (e.g., *beard*). To our knowledge, there have been only a small number of published datasets with norms for semantic gender in Czech (Preininger et al., 2025), Dutch (Vankrunkelsven et al., 2024) and English (Crawford et al., 2004; Scott et al., 2019), although it has been explored using corpus-based methods (see Sap et al., 2014). The dimension has been relatively underexplored in the lexical processing literature, but has been utilized in studies of linguistic relativity (e.g., Brand et al., 2025), where it is of theoretical interest when exploring hypotheses about the interaction between grammatical systems (that formally mark gender by assigning nouns a grammatical gender, e.g., masculine/feminine/neuter) and the conceptual representation of gender information (see Samuel et al., 2019).

### Imageability

This dimension also captures an aspect of sensory experience, but the focus is more on the ease with which an individual can form an internal sensory image of a concept, typically assessed through a continuum of highly unimageable (e.g. *theoretical*) to highly imageable (e.g. *carrot*). Theoretically, the greater the imageability of a concept, the easier it should be to process, in line with dual coding accounts of processing (Paivio, 1990), as highly imageable concepts can utilize both verbal and nonverbal channels. Although the magnitude of imageability effects in lexical processing has been scrutinized (e.g., Dymarska et al., 2023), they have been argued to be a theoretically distinct construct from other sensorimotor-based dimensions, such as concreteness (Kousta et al., 2011), and can be of specific interest for clinical studies where mental imagery is difficult, e.g., aphantasia (Muraki et al., 2023).

### Valence

This dimension captures the extent to which a concept is related to emotional affect, typically through a bipolar scale anchored by negative and positive, with a neutral midpoint (Warriner et al., 2013). Although words can be classified as negative (e.g., *bully*), neutral (e.g., *tepid*), or positive (e.g., *peace*) along the continuum, the extent to which polarity impacts processing has been examined, with items that have high absolute valence being processed faster than neutrally valenced items (Kousta et al., 2009). This is in line with Lang et al.’s (1997) motivated attention account, placing the priority on processing of motivationally significant stimuli, over ones that do not engage reward or aversion.

## Data summaries for norming studies

As outlined in most of the norming studies mentioned in the paper so far, a typical methodological approach has been to use a Likert scale to collect subjective judgments from participants, which are used to capture the underlying construct of the dimension being investigated. However, when studies provide the summative data at the item level, the dominant descriptive statistics that are provided are almost exclusively the mean (as a measure of central tendency that represents the average over all participants) and the standard deviation (as a measure of variation in the data across participants). However, the sensitivity and suitability of such measures have been critiqued. The main criticism is that Likert scales are fundamentally ordinal in nature, i.e., they represent ordered categories, not a continuous metric scale, which can lead to distortions in the way the data is summarized and even introduce higher rates of type I and type II errors (Liddell & Kruschke, 2018). To counteract this, Taylor et al. (2023) demonstrated that, in order to account for participant-level variation and the ordinal structure of Likert scales, rating norms should be calculated using cumulative link mixed-effects models (CLMMs). Instead of using means, CLMMs allow the raw data to be modeled in a way that can utilize random effects, such as participant or item ID, and use them to account for variance in the data, but also as estimates of how those effects vary within the population, thus providing a latent estimate that can be used as the summative norm.

Another benefit of using the CLMM approach is the option to utilize Bayesian methods in the modeling of the estimates, a point highlighted by both Taylor et al. (2023) and Veríssimo (2021), where convergence issues are removed, and flexibility in the complexity of the model can also be increased. Whilst having a traditional mean as the summary variable for a norming database is useful for comparison to existing datasets, often the raw data is not publicly available to derive CLMM-based estimates, so there still remains the question of whether the approaches lead to meaningful differences when validating the norms in lexical processing tasks.

## Aims of the present study

The core aims of the present study are:

1. To make openly available a novel dataset that provides normative estimates for a large set of Czech words with good validity, along with the raw participant-level response data and basic demographic information for each of the participants.
2. Validate the resulting norms through a series of analyses that use them as predictors in regression models predicting performance on a lexical decision task focusing on accuracy and reaction times (RTs).
3. Assess the extent to which different aggregation methods – traditional means or CLMM-derived latent means – provide any differences in the outcomes when predicting lexical processing.
4. Outline potential future uses of the norms as a large-scale gold-standard human-derived dataset.

## Method

### Participants

A total of 4626 participants were originally recruited to participate in the study using a university-wide participant pool at Charles University, located in Prague, Czechia. Participants took part for course credit. We subsequently filtered out any participants who reported that Czech was not their native language (*n* = 217), in addition to any participant who reported being under the age of 18 years (*n* = 3). One participant was further removed on the basis of quality checks; see Data filtering section below. The final participant sample comprised 4405 participants (3350 females, 1022 males, 23 non-binary, 10 undisclosed, median_age_ = 21, SD_age_ 4.73, range_age_ = [18:62]). Ethical approval was granted by the Charles University ethics committee.

### Stimuli

Our original stimuli set comprised 9723 items, which spanned a range of different parts of speech. The items were derived from two different sources: firstly, the HeCz corpus (Chromý et al., 2025), which sourced newspaper headlines from a popular Czech newspaper website https://www.seznamzpravy.cz/ for a self-paced reading corpus, and secondly, the SocioLex database (Preininger et al., 2025), which is a large socio-semantic norming study on Czech. After a number of data cleaning steps were applied to the raw data (see subsection below), our final dataset contained a total of 9601 items for which we had at least ten ratings for a dimension.^2^ There were 1745 adjectives, 5713 nouns, 1462 verbs, and 681 items that had a different part of speech (such as adverbs, prepositions, numerals, conjunctions); see Chromý et al. (2025) for details on the morphological annotation.

### Procedure

The experiment was programmed using jsPsych (de Leeuw et al., 2023) and was accessible online via a browser, with a JATOS server being used to host and store the data (Lange et al., 2015). We set up the experiment so that participants could only complete it using Google Chrome on non-mobile devices; this was to ensure that there were no technical differences across participants in the way the experiment was presented visually. All code and scripts for the experiment are available on the OSF repository. Participants first read a detailed instruction page, provided informed consent, and completed a brief demographic questionnaire. Participants were then asked to rate each of the words from a randomly selected list (each starting with a calibrator word followed by a randomly ordered set of 121 or 122 words). Each list contained a range of words with different parts of speech; specifically, we ensured that each list would contain 18/19 adjectives, 70/71 nouns, 20/21 verbs, and 11/12 items that had a different part of speech. Participants rated a different list of words for each of the dimensions, with all participants being presented with all five of the dimensions during the experiment, but the lists were always randomly assigned within participants, so they never completed the same list of words across dimensions. There were 80 different lists of words in total.

Within each list, there was also a control word that was presented randomly with the other items. The calibrator words were included to ensure that participants were using the scale as intended at the very start of rating each dimension, whereas the control words were included primarily as a quality check within each dimension to ensure participants were rating the items in a suitable manner. See the data cleaning section for more details on this. Both the calibrator and control words were selected on the basis of them having reliable ratings with low variance in a pilot version of the experiment. See Table 2 for details of what the calibrator and control words were for each of the dimensions.

**Table 2:** Dimensions that have been normed, with the types of scales used in the experiment and the anchors used for the scale. The calibrator and control words are also given for each of the dimensions, along with example items that have mean values across the span of the scale. English translation equivalents are given in square brackets. The median number of participants who rated items is given in the final column, along with the median number of ratings based on how many participants reported knowing the item

Dimension	Scale	Anchors	Calibrator/control	Examples	Median *N* ppts (median *N* known)
AoA	Free response numeric	NA	pejsek [*doggy*]/ federalismus [*federalism*]	máma [*mommy*] (1.63), bota [*shoe*] (3.03), plavčík [*lifeguard*] (6.58), evakuace [*evacuation*] (8.81), badatel [*researcher*] (10.19), patologický [*pathological*] (14.26), bitcoin [*bitcoin*] (18.06)	35 (33)
Concreteness	5-point Likert	1 = abstract 5 = concrete	teorie [*theory*]/ neoliberalismus [*neoliberalism*]	jestli [*if*] (1.1), rozum [*reason*] (1.88), úplně [*completely*] (2.20), jemný [*fine*] (3.14), francouzština [*French*] (3.79), kresba [*drawing*] (4.33), bunda [*jacket*] (5.0)	35 (33)
Gender	7-point Likert	−3 = very masculine 3 = very feminine	náhrdelník [*necklace*]/ otec [*father]*	varlata [*testicles*] (− 2.71), mnich [*monk*] (− 2.04), bořit [*to demolish*] (− 0.94), sůl [*salt*] (0.01), červenat se [*to blush*] (1.08), zdravotnice [*nurse*] (2.06), paní [*lady*] (2.85)	40 (38)
Imageability	7-point Likert	1 = difficult to imagine 7 = easy to imagine	orel [*eagle*]/ chipsy [*crisps*]	však [*however*] (1.55), zda [*whether*] (2.03), možný [*possible*] (3.04), apatie [*apathy*] (3.97), autismus [*autism*] (5.00), komik [*comedian*] (6.05), brambor [*potato*] (7.00)	34 (33)
Valence	7-point Likert	−3 = very negative 3 = very positive	šikanovat [*to bully*]/ štestí [*happy*]	týrání [*abuse*] (− 2.93), epidemie [*epidemic*] (− 1.94), truc [*spite*] (− 1.03), kapuce [*hood*] (0.02), mýdlo [*soap*] (0.99), radovat [*to rejoice*] (2.08), šťastný [*happy*] (2.88)	40 (37)

**Fig. 1:**
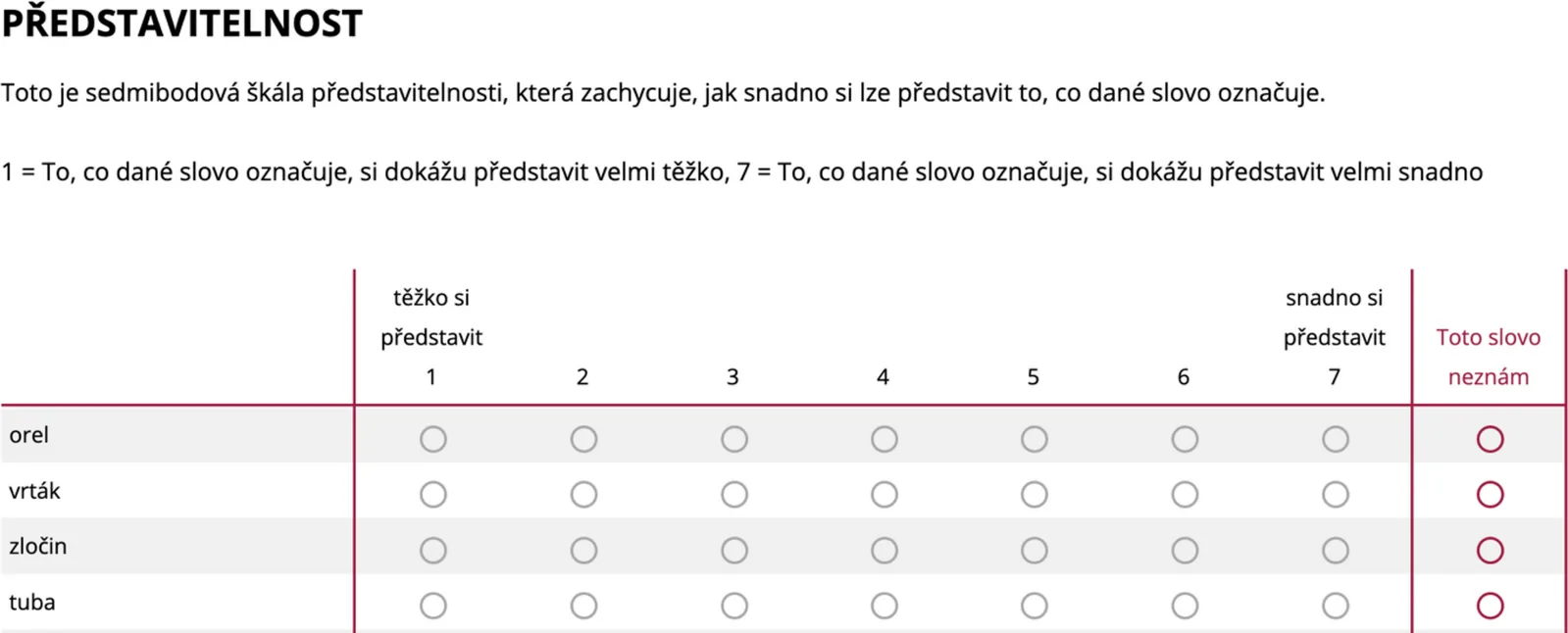
Example screenshot of the experimental setup for the imageability rating part of the experiment. The words being rated are on the right-hand side, with the scale being anchored by ‘těžko si představit’ [difficult to imagine] and ‘snadno si představit’ [easy to imagine], with the option to choose ‘toto slovo neznám’ [I don’t know the meaning of the word’] in the rightmost column. The instructions are presented at the top of the screen; see the OSF repository for a full list of instructions along with their English translations

The instructions given to participants for each dimension, together with their English translations, can be found on the OSF repository, with a screenshot for the imageability dimension provided in Fig. 1. Participants were presented each dimension sequentially, with the order of presentation for dimensions and items being randomized for each participant (apart from AoA, which was always the final dimension to be presented, and the calibrator words, which were always the first to be presented). All dimensions included an additional option for each item where participants could choose “*toto slovo neznám”*, indicating that they did not know the meaning of the word, so no rating for that item was provided.

### Data cleaning

**Fig. 2:**
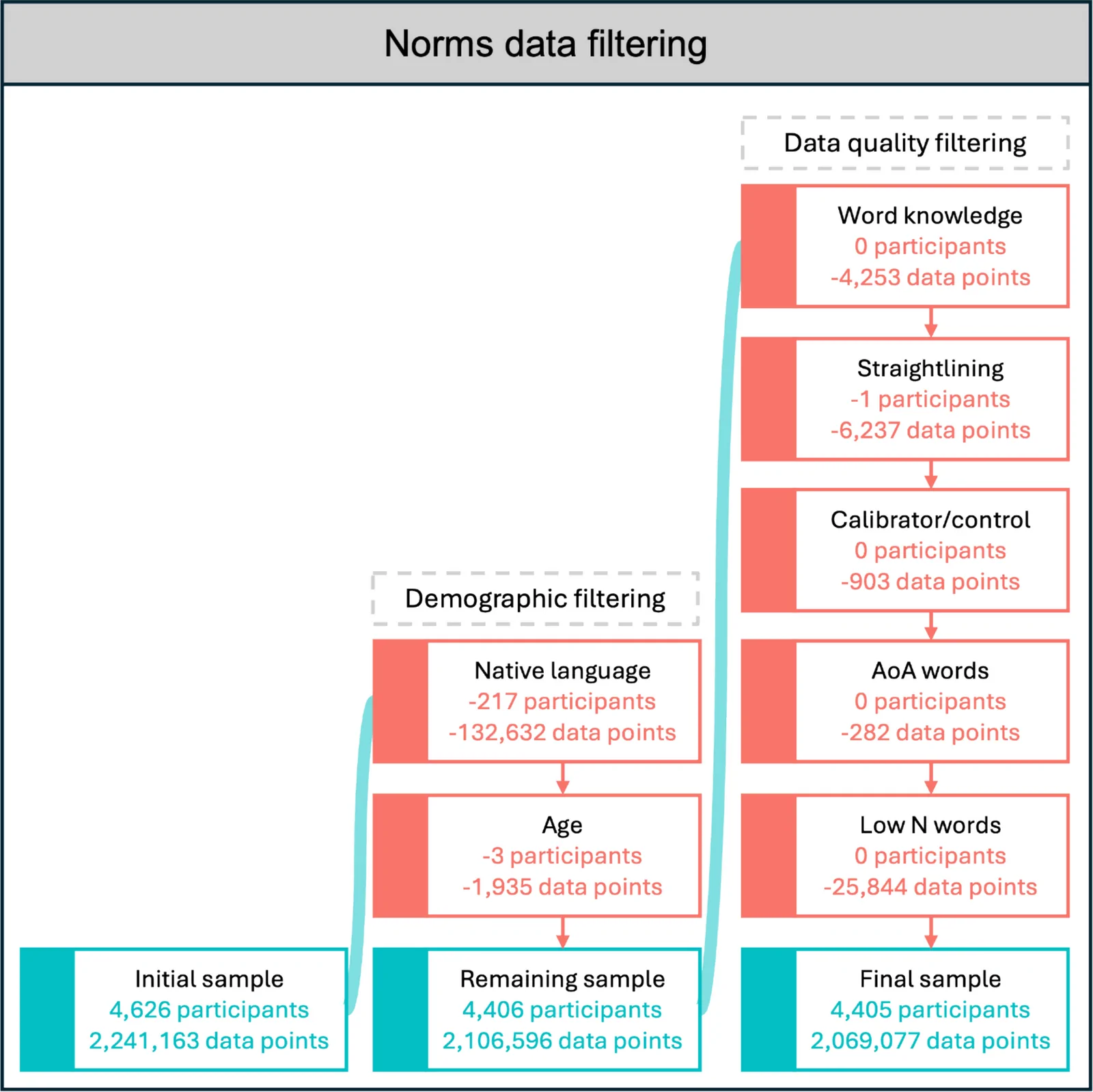
Filtering procedure used on the raw data. *Turquoise boxes* contain summaries of the data that remain after each filtering block; *red boxes* contain summaries of the data removed during each filtering step

All cleaning, processing, and analyses reported below were conducted using R version 4.5.2 (R Core Team, 2025). All the code and data (raw and processed) can be found in the OSF repository. Following the initial demographic filtering of native language and age, we carried out a number of data quality checks to ensure that we detected any low-quality responses/participants; this process is visualized in Fig. 2. The code used to run these checks can be found in the OSF repository.

1. *Word knowledge:* Across the whole sample, the mean number of items reported to be unknown by participants was very small at ~ 4%. Therefore, we decided to remove data on a per-dimension basis, whereby any participant who reported not knowing > 25% of the items for a given dimension would have the data for that dimension removed, which resulted in the removal of 4253 rows of data. Although no individual participant was completely removed as a result of this, 33 participants were affected.
2. *Straightlining:* We inspected the variance in the participants’ ratings to identify ‘straightliners’, i.e., participants who selected the same response for the majority of the items (Kim et al., 2019; Winter et al., 2024). If a participant straightlined on more than 95% of the items for a dimension, we removed the data for that participant on that dimension. This led to the removal of 6237 rows of data affecting 45 participants, including the complete removal of one participant.
3. *Calibrator/control words:* We analyzed the responses to the calibrator and control words for each of the five dimensions to ensure that participants could demonstrate that they were using the rating scales as expected. For each calibrator/control word, we identified participants who responded to the word on the opposite side of the scale we expected. For example, the word *štěstí [happiness]* for the valence dimension was expected to have a response somewhere on the positive side of the scale, so if a participant responded to this word as negative in any way, this would be flagged. Any participant who responded to both the calibrator and control items in the opposite direction to our original expectations would have their data for the dimension removed. This resulted in 903 rows of data being removed, affecting seven participants, although no participant was removed completely.
4. *AoA responses:* In order to address some issues with the free response data on the AoA dimension, we conducted the following filtering steps. If a participant provided an age that was higher than the reported age, e.g., saying a word had an AoA of 28 when they reported being 20, we removed this data at the item level. Similarly, we also removed any data containing negative values, e.g., – 5. If any participant had more than ten items where either of the first two conditions were flagged, we removed their data for the AoA dimension altogether. These steps led to the removal of 282 rows of data, affecting 23 participants, but no participant was removed from the data completely.
5. *Low N words:* In order to ensure each item had sufficient data to obtain a reliable estimate for the dimension, we applied a filter to remove any item with < 10 responses (as was the case in Winter et al., 2024). This resulted in the removal of 25,844 rows of data, with 352 items affected. Although an item may have a low *N* for a specific dimension, we did not remove the item completely if it had sufficient *N* ratings on other dimensions.

### Additional lexical and processing variables

To supplement our dataset, we also provide a number of additional variables that researchers may find useful for additional analyses or to control for when choosing stimuli. As these are not readily available for Czech, but are used in subsequent analyses within this paper, we will provide a brief overview of how these variables were computed or collected. However, we would like to highlight that these variables are not the core focus of the current paper, and we would like to direct readers to Brand et al. (in prep) for a more focused write-up related to them.

#### ZipfFrequency

Using the SYN2025 corpus of Czech from the Czech National Corpus (Křen et al., 2025), we extracted frequency counts for each item in our corpus, excluding any bigram items. This returned data for 9316 items for which there was a frequency count greater than or equal to 1. We then manually coded any remaining unigram items as having a frequency of 0, which applied to 161 items. These values were then converted to a Zipf frequency score (Van Heuven et al., 2014).

#### OLD20

This is a measure of orthographic Levenshtein distance, where the values represent the average edit distance in order to transform the item into its 20 nearest neighbors, with low values representing dense neighborhoods, and large values representing sparser neighborhoods (Yarkoni et al., 2008). The corpus used to calculate this was the Phonological Corpus of Czech (Bičan, 2013), which contains phonological coding for 275,779 words. OLD20 values were calculated using the *vwr* package in R (https://github.com/cran/vwr).

#### nchar

The orthographic length of the item. This was calculated as the string length in characters, ignoring any whitespace.

#### rt_intercept

In order to acquire an estimate of lexical processing time, we used reaction times from a large lexical decision task (Brand et al., in prep; for another comparative dataset, see also Chromý et al., 2026). The design followed a standard lexical decision paradigm where participants were asked to respond to orthographic word forms on the basis of whether they believed the word to be a real Czech word or not by pressing a button on a keyboard. The experiment was conducted online, and participants were recruited from the same university participant pool as used in the current paper, so their demographics were closely matched for age and gender. Using the raw response data for reaction times (RTs) where participants responded accurately to the item, we ran a linear mixed-effects model, with a global intercept and random intercepts for participant and item, meaning there were no fixed effects included as we did not wish to control for any predictor variables. We then extracted the random intercepts for the item variable, which are mean-centered around 0, with lower values representing faster processing times and higher values representing slower processing times. This approach was preferred over using just the standard mean aggregation, as the values do not need to be log-transformed, and they account for other sources of variation at the participant level; see Drager and Hay (2012) for more details on this.

#### acc_intercept

Using the same data as was used in the calculation of the *rt_intercept* values, we estimated the accuracy of responses to the items using a generalized linear mixed-effects model, predicting whether the participant had an accurate response (coded as 1) or an inaccurate response (coded as 0). We use the same basic model with random intercepts for participant and item, and extract the intercepts for the item variable. These values were then used as estimates of accuracy for each item, with lower values representing lower accuracy and higher values representing greater accuracy.

### Analysis and discussion

Our analysis will focus on a range of different approaches to demonstrate the way the raw data can be used for: 1) providing a general description of the data, 2) exploring the relationships between the dimensions and linguistic variables, 3) aggregation into summary estimates, 4) assessing the validity of the summary estimates when predicting measures of lexical processing. Prior to all analyses, we converted the rating data into numeric values, with all responses that were coded as the participant not knowing the meaning of the word as NA values. All analyses reported were conducted in R version 4.5.2, with all scripts available on the OSF repository.

### Descriptive statistics

#### Reliability

In order to assess whether there was good reliability in the ratings for the items, we analyzed the raw data using an intraclass correlation coefficient (ICC). This was achieved by using the ICC2.lme function from the *psychometric* package. This was set up as a linear mixed-effects regression model, predicting the rating value by a random intercept for item and a global intercept as a fixed effect (see Brysbaert, 2019). These were run separately for each dimension, with the results indicating very good reliability across all dimensions: AoA =.98; concreteness =.96; gender =.97; imageability =.93; valence =.97.

#### Traditional data summaries

**Fig. 3:**
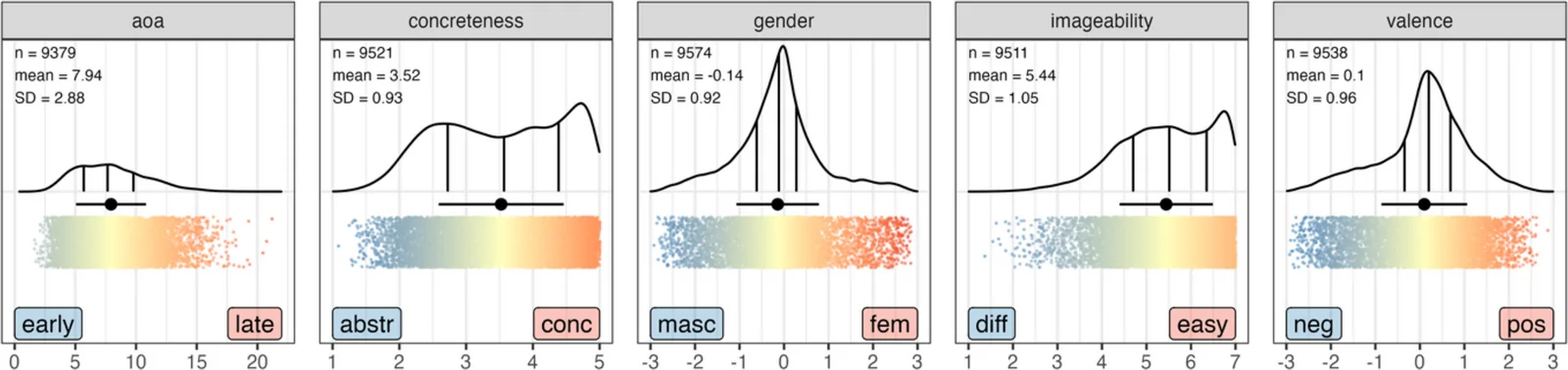
Distribution of mean ratings for the AoA, concreteness, gender, imageability, and valence dimensions. Kernel density estimates are shown with 25%, 50%, and 75% quantiles marked by *solid vertical lines*. The *black point with a horizontal line* represents the mean and standard deviation of the ratings. Each word is represented by a colored point, with more *red/blue colors* indicating a stronger association towards a specific side of the scale, and *yellow points* associating more with the mean across all ratings

As a first step in the analysis of the ratings, we calculated the standard descriptive statistics for each item across each dimension. This included the number of participants who rated the word (*n_sample*), the number of participants who provided a rating, i.e., not reporting they did not know the meaning of the word (*n_known*), the proportion of responses that were given, i.e., n_sample/n_known (*prop_known*). We also calculated basic summary estimates that aggregated the ratings to give the mean and standard deviation (SD) (see Fig. 3 for a visualization of the aggregated data and Table 2 for examples of words across the range of values for each dimension). As an additional check of validity, we compared our ratings to those from previous studies on Czech using a Pearson’s correlation analysis, specifically for AoA (Łuniewska et al., 2019), imageability (Smolík, 2019), gender, and valence (Preininger et al., 2025); all correlations were found to be very strong (all having *r*’s >.87 and *p*’s <.001), indicating no major deviation from previous datasets in Czech.

### Relationships between variables

As an exploratory analysis, we decided to explore the correlations between the variables we have collected data for, in addition to a number of lexical variables related to each item. Although not underpinned by any specific theoretical motivation, we were expecting trends in the data that have been previously reported, e.g., a strong correlation between concreteness and imageability, and AoA to be strongly correlated with word frequency.

**Fig. 4:**
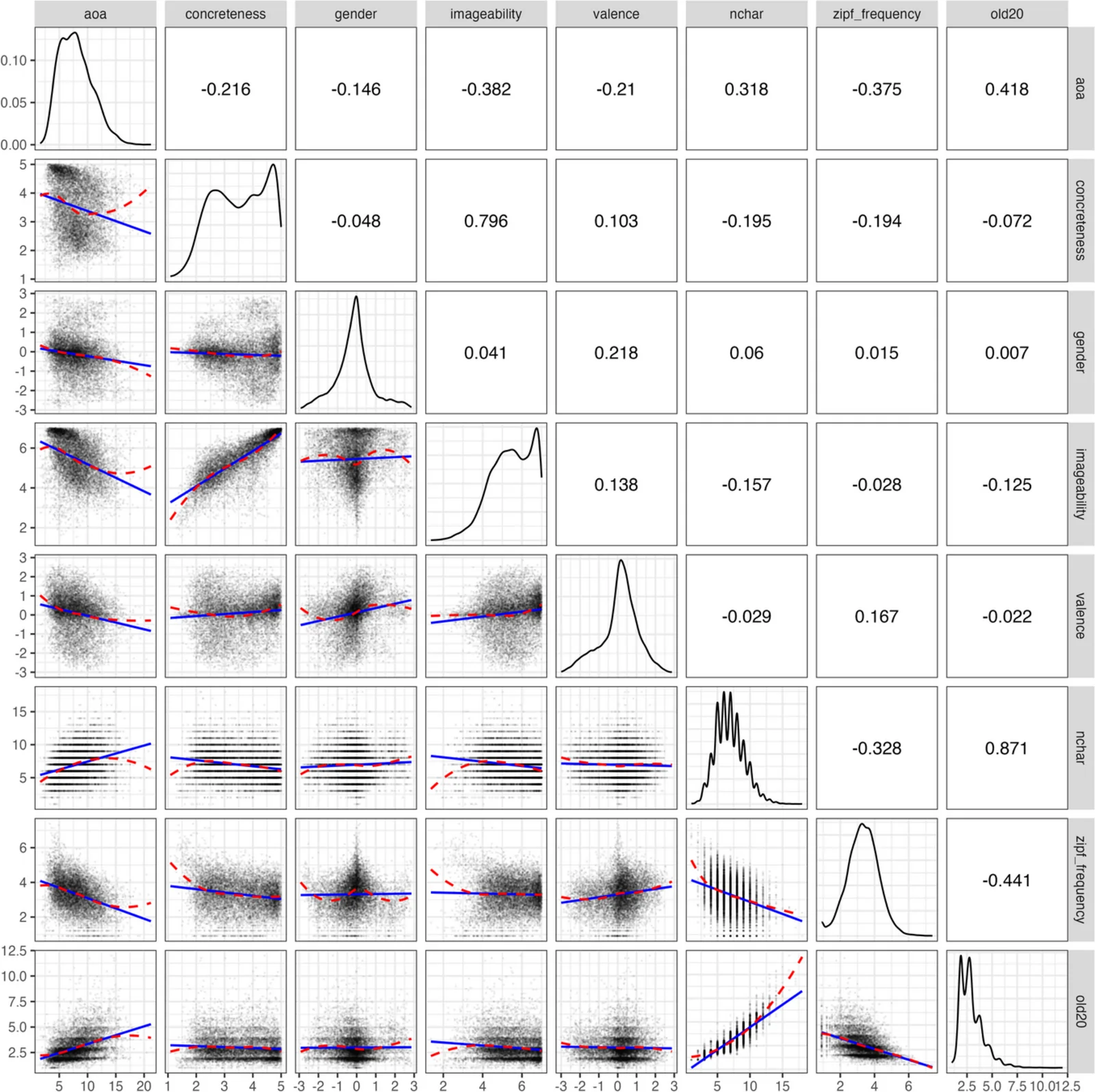
Correlation matrix of all the dimensions in the word ratings dataset. The *lower left plots* are scatter plots of the data fitted with a linear (*blue*) and LOESS (*red*) fit, with the data from the dimension in the top facets on the *x*-axis and the data from the right facet on the *y*-axis. The diagonal plots show kernel density distributions of the variables. The *upper right plots* give the Pearson correlation coefficients between two dimensions

The results of the correlation analysis are visualized in Fig. 4. As this was an exploratory analysis, we do not provide any null hypothesis significance testing, and simply report the Pearson’s correlation coefficients to be used as a descriptive indicator of relationship strength. Figure 4 also presents scatter plots of the data, with linear and non-linear fits, which indicates that some of the relationships may be better fit with non-linear models.

Given the number of correlations presented, we will not report on each individual relationship. However, there were a number of particularly strong linear relationships that can be observed. Concreteness and imageability had the strongest relationship within the five dimensions (*r* =.796), a pattern that has been well documented in previous studies; Scott et al., 2019, with Scott et al. reporting *r* =.91), while more modest relationships were found between gender and valence (*r* =.218), replicating the finding in Scott et al. (2019) that more feminine words were evaluated more positively (with their study reporting *r* = –.42, although their gender scale was in the reverse direction to the present study). Likewise, there was also a negative relationship between AoA and imageability/concreteness (*r* = –.382 and –.216, respectively), with more imageable/concrete items being acquired earlier, which again was reported in Scott et al. (2019) analysis (with their study reporting *r* = –.49 and *r* = –.38, respectively). We do note that compared to Scott et al.’s results, the correlations reported here are evidently slightly smaller in size; this may be as a result of a much larger set of items that cover a more diverse lexical inventory. Nevertheless, the patterns are consistent across the English and Czech analyses.

### Aggregation methods

#### Cumulative link mixed effects models

In the Descriptive statistics section, we reported norming summaries using the mean, which is a typical approach used when aggregating rating data from Likert scales, treating the individual item and participant-level ratings as integer values (e.g., very negative on our valence scale coded as – 3). However, this approach may not be an optimal way to aggregate such data, as means fail to respect the ordinal nature of Likert scales (see Liddell & Kruschke, 2018; Veríssimo, 2021). Following the recommendations of Taylor et al. (2023), we instead modeled participant responses using CLMMs implemented in the *ordinal* package (Christensen, 2018) in R. These models account for variation introduced by participant-specific response biases and thus yield a more accurate estimate of the normative value for each item. For each dimension, we fitted a separate model predicting participant responses (coded as an ordinal factor, e.g. for valence, – 3 < – 2 < – 1 < 0 < 1 < 2 < 3) with random intercepts for both item and participant. From these models, we extracted the random intercepts for each item, which provided numeric estimates of the latent means across each dimension.

#### Bayesian models

As an additional aggregation method, Taylor et al. (2023) also demonstrated that these models can be fit in a Bayesian framework using the *brms* package (Bürkner, 2017; see also Bürkner & Vuorre, 2019), where latent means for items can again be estimated. An additional benefit outlined by Taylor et al. (2023) for using this approach is the ability to estimate a latent measurement of variance in the ratings, which are estimated from ordered regions of the Likert scale, contrasting with the traditional metric of calculating a standard deviation (SD) that is derived from a continuous representation of the Likert scale. A core argument against using the SD as a measure of variance with Likert scale responses is that meaningful differences in the way participants respond to the item being rated, for example, when rating ambiguous or polysemous items (Filipović Đurđević & Kostić, 2017) or smaller variance at the ends of the scales, can be conflated with response biases introduced by the participants. To overcome this, Taylor et al. demonstrate that modeling the ordinal ratings using a Bayesian framework allows for the estimation of a latent SD, enabling meaningful variation to be captured, with less influence from participant-level artifacts.

#### Comparison of methods

In the analysis below, we explored the extent to which the different methods of estimating the norms may result in different patterns for item-level values. To do this, we first used the traditional summary statistics reported in the Descriptive statistics section for the mean and SD. Next, we ran CLMMs for each dimension, extracting the item intercepts from each model, which we will refer to as ‘*clmm’* values. Finally, we fitted CLMMs using a Bayesian framework with brms models, with the same structure as the clmm method, but with the addition of a discrimination parameter that was used as a predicted variable, with the same random effects structure (see Taylor et al., 2023 for more details or the OSF repository for full model specifications). This method gave us both a latent mean, which we will refer to as ‘*brms’* values, in addition to a latent SD, which we will refer to as ‘*latent SD’*. Note that we did not run models for AoA, as the nature of the data was not taken to be ordinal, as participants were asked to provide a numeric estimate of the AoA values, not through a Likert scale response. As we are not specifically interested in hypothesis testing within this analysis, we will only provide a descriptive overview of the trends in the data and not report any null-hypothesis significance testing.

#### Item estimates

**Fig. 5:**
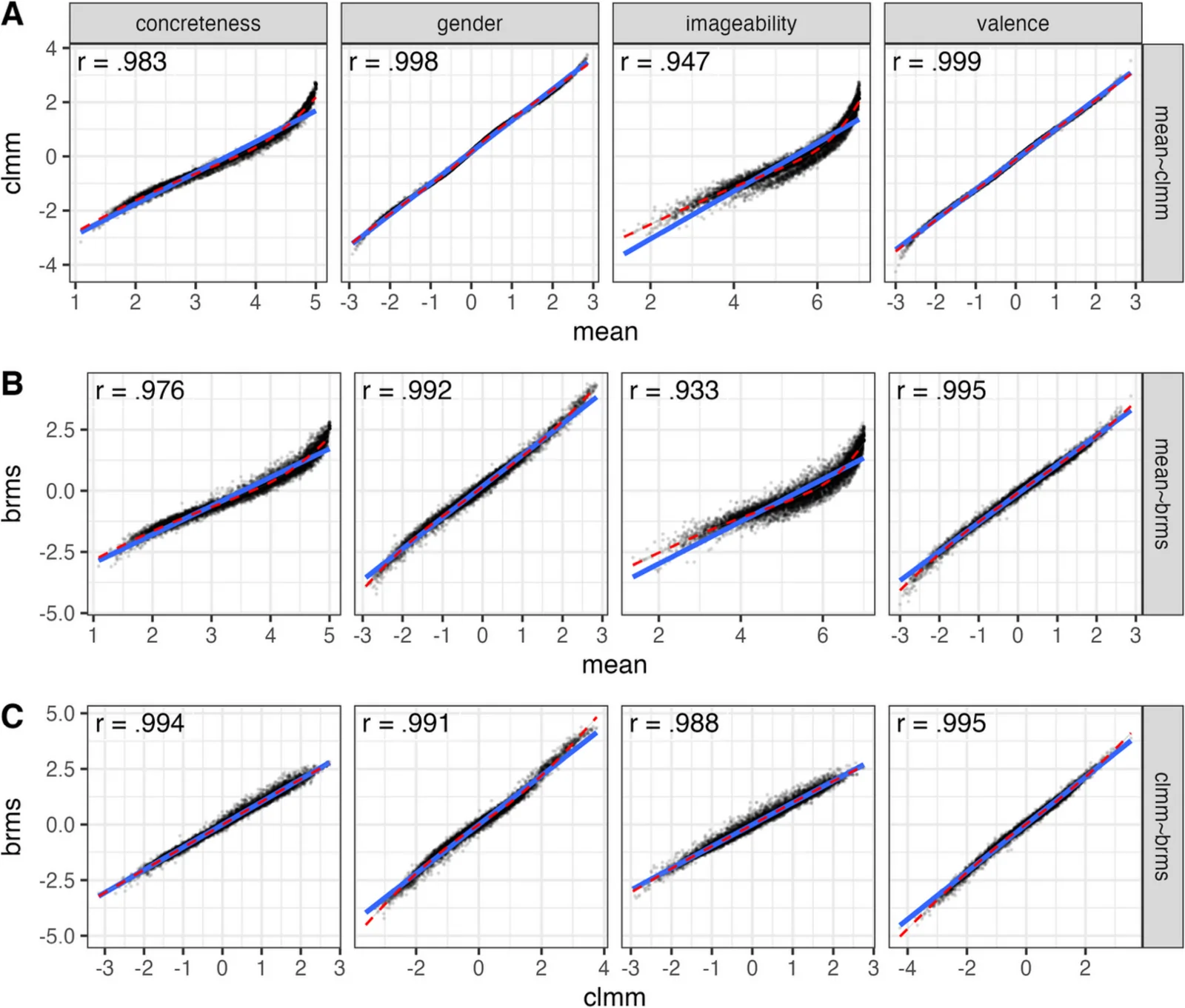
Relationships between the three different aggregation methods across four different dimensions (top facets), with **A** giving the traditional mean on the *x*-axis and the clmm estimates on the *y*-axis, **B** giving the traditional mean on the *x*-axis and the brms estimates on the *y*-axis, and **C** giving the clmm estimates on the *x*-axis and the brms estimates on the *y*-axis. The *blue lines* provide a linear fit to the data, and the *red dashed lines* a LOESS fit. The correlation coefficients are given in the top left corner of each sub-plot

As we can see from Fig. 5, there is generally strong alignment across all the different approaches when estimating the item norms, with Pearson’s correlation coefficients all being *r* >.93. This indicates that even though there was variation in the estimates across items for the different approaches, there is generally a very strong linear relationship. The most salient difference was observed when comparing the data within the imageability dimension, where there is a slight cubic non-linear relationship when the clmm and brms estimates are compared to the mean (plots A and B of Fig. 5). This is also observed, in a weaker way, with the concreteness data, where the imageability means there is a skewed distribution towards the higher values of the scale. However, when comparing the clmm and brms estimates (plot C of Fig. 5), we can observe no distinct differences in the relationships, indicating that the two estimates are very strongly related across all dimensions.

#### Item variance

**Fig. 6:**
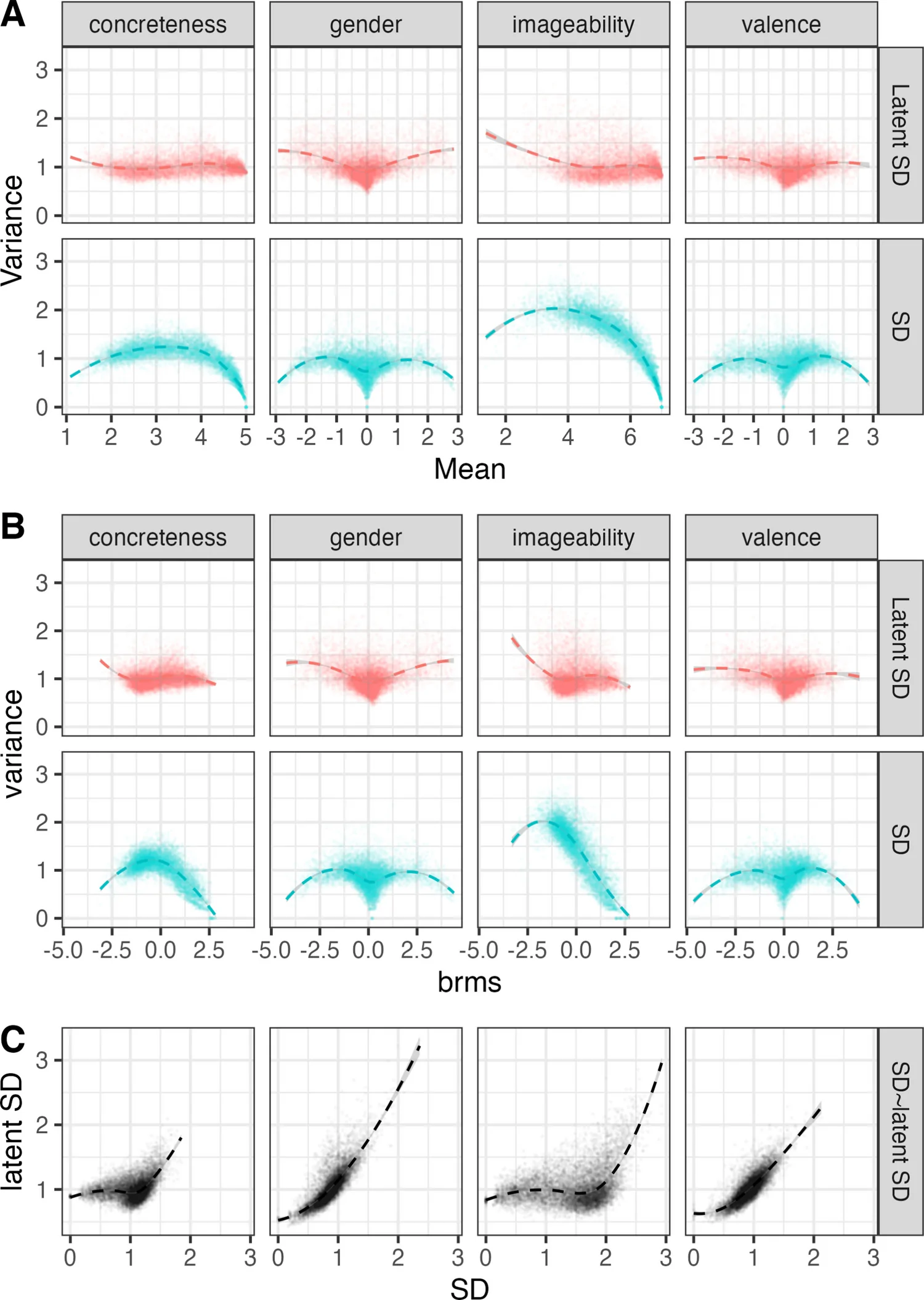
Relationships between the different measures of variance across four different dimensions (top facets), with **A** giving the traditional mean on the *x*-axis and the variance based on the traditional SD and latent SD on the *y*-axis. **B** giving the same relationships as A, but with the brms estimates on the *x*-axis. **C** gives the relationship between the traditional SD and the latent SD. All of the plots are fitted with LOESS smooths

As we can see from Fig. 6, there are some differences in the relationships between the item estimates (Fig. 6A using the mean and Fig. 6B using brms) and the way variance was estimated using the traditional SD (turquoise data) and the latent SD (red data) from brms modeling. As expected, there is smaller variance at the endpoints of the bounded ordinal scales of gender and valence, with the majority of ratings around the neutral midpoint, where variance for items is smaller; the greatest variance is observed for intermediate points between the poles of the scales and the midpoint. This differs from the scales of imageability and concreteness, where there are many data points for larger values, i.e., concrete/easy to imagine, but fewer at the lower end of the scale, i.e., abstract/hard to imagine, resulting in smaller variance when the aggregate values are larger.

The trends for the relationship between the mean and SD appear to be similar to previously reported patterns, i.e., smaller SD at the largest values of the scales for concreteness and imageability, whereas the polar values for gender and valence have smaller SD values, with the greatest spread in SD values around the neutral mid-point. These patterns are also seen when using the brms estimates, which is unsurprising given the strong correlation of those values with the mean.

When inspecting the relationships between the latent SD and estimates using the mean and brms values, we observe a similar pattern to that reported by Taylor et al. (2023), there is no longer a distinct trend for extreme values to have lower variance. This suggests that the approach reduces the artifact of response biases and instead preserves the meaningful variability that exists within the ratings. For variables where there is a neutral mid-point – gender and valence – there still remains a similar, but perhaps weaker, trend as observed for the traditional SD, i.e., the items with a value around the midpoint have the largest spread of latent SD values. This could indicate that items that have a norm that has been estimated to be around the neutral midpoint may simply have a very uniform rating, where most raters identify it as neutral, whereas other items may be averaged over due to meaningful variation across raters Chromý et al. (2025).

### Predicting measures of lexical processing

In order to assess the validity of the norms, we provide an analysis that aims to assess the extent to which measures of lexical processing can be predicted by the normative estimates across the different dimensions. To do this, we modeled data using a hierarchical regression approach predicting values from a lexical decision task, focusing on the reaction times (RT) and response accuracy for the items (see Sect. 3 for a description of these variables). This is a standard procedure to assess the validity of semantic norms, and each of the variables has previously been reported to have significant effects in other languages (e.g., AoA: Kuperman et al., 2012; concreteness: Bonin et al., 2018; gender: Preininger et al., 2025; imageability: Khanna & Cortese, 2021; valence: Kousta et al., 2009). In total, we had complete cases for 9243 items, and all analyses reported in this subsection use this dataset.

As a first step, we created baseline models for both RT and accuracy using a simple linear regression with predictor variables of Zipf frequency, orthographic length, and OLD20. After assessing the variance inflation factors (VIFs) for the models, there was a potential issue with including both orthographic length and OLD20 (VIFs = 4.44 and 4.90, respectively), so we decided to exclude the orthographic length variable as OLD20 has been reported to be a more theoretically interesting variable (Yarkoni et al., 2008). The baseline model was used to account for variance explained by linguistic variables, which are known to be reliable predictors of lexical processing.

Our next modeling step included the psycholinguistic variables collected in the current study – AoA, concreteness, gender, imageability and valence. All of these variables were treated as continuous values; however, we did transform the valence variable into absolute values to ensure that polarity was not a factor (Kousta et al., 2009). After inspecting the VIFs for the models, we found that both concreteness and imageability had values around 4.00 (concreteness = 3.92 and imageability = 4.00). As the two dimensions are also theoretically capturing similar constructs, we decided to drop the concreteness variable from the analyses to avoid any potential sources of collinearity and because imageability effects are more consistent in analyses of lexical decision measures (Connell & Lynott, 2012).

We ran three separate versions of these models using the estimates from the mean, clmm, and brms methods. This allows us to then compare whether the different methodologies of aggregating the norms provide differing results. Although we did not predict any major differences, we did want to explore this as an additional level of analysis.

For reference, as we were using measures for RTs and accuracy derived from mixed-effects modeling, instead of the traditional descriptive statistic of the mean, we conducted parallel analyses of all the results reported below and found no indication that the two approaches led to any differences in the pattern of results; indeed, they were almost identical in terms of the general patterns being reported. This lack of a difference between the results may be explained by the fact that our analysis had very large samples at the participant and item level, so variance within the intercepts and traditional mean measures would consequently both be low, compared to an analysis with smaller samples. Nevertheless, it is important to highlight that the use of extracting random intercepts for a secondary analysis, and the added complexity involved when using them in this way, needs to be justified.

In our analysis, we focused on the intercept-derived estimates as our sample sizes were sufficiently large. Additionally, our semantic norms were also derived from intercept-derived estimates, where participant-level variation was being considered in the models, so this provided us with a unified approach across our estimates. The practice of extracting random intercepts for analyses has been suggested to be a useful approach when considering alternative ways of obtaining item-level estimates for analyses (see e.g., Drager & Hay, 2012); however, there are also reasons to be cautious about the approach. For example, Houslay and Wilson (2017) and Kliegl et al. (2011) both discuss the limitations of using this approach, as the estimates do not carry forward the error from the estimates themselves, which can lead to an increased likelihood of obtaining false positives in subsequent analyses that use the intercepts. While the results from both the intercepts and traditional mean do not appear to represent any substantial divergence, future work may investigate the conditions under which different methods of obtaining estimates may, or may not, lead to different outcomes in the analyses.

#### RTs

**Fig. 7:**
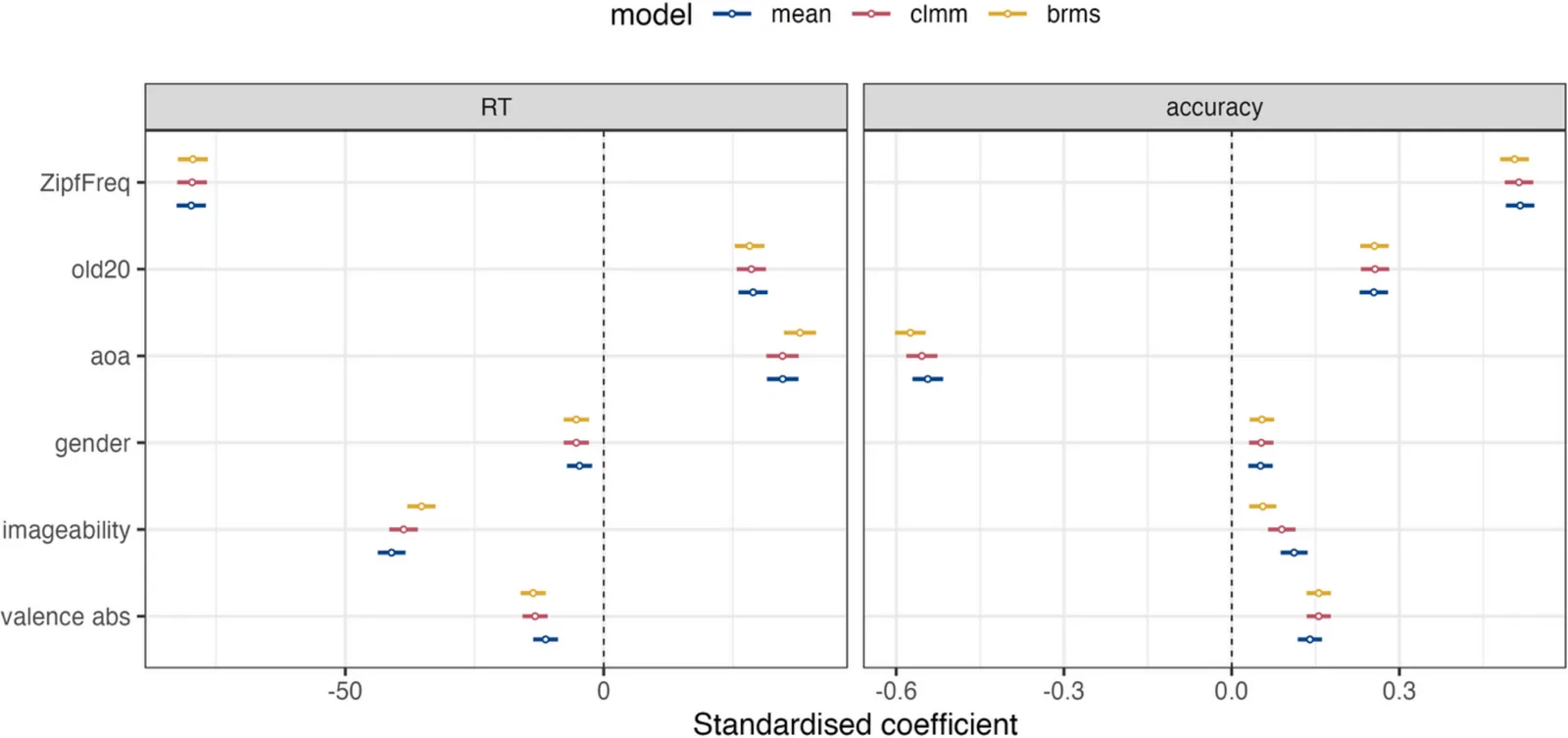
Model coefficients and 95% confidence intervals when predicting data from the lexical decision task. The *x*-axis provides the standardized coefficients, with the predictor variables given on the *y*-axis. The left facet visualizes RTs and the right facet visualizes accuracy. The colors represent the different models from which the values for each predictor variable were derived (*blue* = mean, *red* = clmm, and *yellow* = brms)

The baseline model had an adjusted *R*^2^ =.411, with both Zipf frequency and OLD20 being significant predictors of RTs. In the second step, all three different models significantly improved the model fit (all *p*s <.001), with adjusted *R*^2^ =.541 and Δ*R*^2^ =.130 for the model with the mean values, adjusted *R*^2^ =.533 and Δ*R*^2^ =.122 for the model with the clmm values, and adjusted *R*^2^ =.527 and Δ*R*^2^ =.116 for the model with the brms values. All of the variables added in this step were significant; see Fig. 7 for the standardized coefficient for each of the variables across each of the models.

#### Accuracy

The baseline model had an adjusted *R*^2^ =.197, with both Zipf frequency and OLD20 being significant predictors of RTs. In the second step, all three different models significantly improved the model fit (all *p*s <.001), with adjusted *R*^2^ =.381 and Δ*R*^2^ =.184 for the model with the mean values, adjusted *R*^2^ =.380 and Δ*R*^2^ =.183 for the model with the clmm values, and adjusted *R*^2^ =.378 and Δ*R*^2^ =.181 for the model with the brms values. All of the variables added in this step were significant; see Fig. 7 for the standardized coefficient for each of the variables across each of the models.

The pattern of results across each of these analyses aligns with previously reported findings. For the effect of AoA, items that were acquired earlier were processed faster and with higher accuracy than items that were acquired later. For gender, items that were more feminine were processed faster and with higher accuracy than items that were more masculine. For imageability, items that were easier to imagine were processed faster and with higher accuracy than items that were harder to imagine. For absolute valence, items that were either more positive or negative were processed faster and with higher accuracy than items with neutral valence. Although there are some minor differences in the amount of variance explained by the different aggregation methods, we would be hesitant to interpret this as clear evidence in support of using one aggregation method over others.

## General discussion

The present paper has introduced the largest database of psycholinguistic normative ratings for Czech words currently available for researchers, covering a multidimensional space of five distinct variables – AoA, concreteness, imageability, gender, and valence. Our first aim was focused on ensuring the dataset was transparent, accessible, and had good reliability, which was addressed through a transparent data processing procedure, making both the raw and summary data freely available to those who wish to use it, as well as including all analysis code so that the analyses can be reproduced easily. Our analysis has presented a range of descriptive statistics within each of the individual variables we collected data for, in addition to exploring the relationships between each of the variables. The distribution of ratings differed depending on the specific dimensions, with concreteness and imageability having a skew towards higher values (more concrete/imageable) and AoA having a skew towards lower values, whereas gender and valence had a much more normal distribution with ratings typically around the neutral mid-point. Such patterns are quite typical and have previously been observed for these dimensions in previous norming studies; see, for example, Scott et al. (2019).

Our second aim related to assessing the validity of the norms, which was addressed through an analysis of lexical processing measures obtained through a lexical decision task (RTs and accuracy). The results of the analysis revealed expected effects for all of the variables, with the model explaining more variation than a baseline with just linguistic predictors included. Specifically, we observed that items that were acquired earlier, more feminine, more imageable, and had a larger absolute valence all had faster RTs and higher accuracy within a lexical processing experiment, aligning with results from previously reported analyses in other languages (e.g., Khanna & Cortese, 2021; Kousta et al., 2009; Kuperman et al., 2012; Preininger et al., 2025).

Our third aim was focused on investigating differences across different aggregation methods, with an assessment of item summary estimates and variance being the key measures of interest. Using the raw rating data, we calculated estimates from basic descriptive statistics (the mean and SD) as well as latent values from ordinal mixed-effects modeling using frequentist maximum likelihood to obtain latent means (clmm), as well as using a Bayesian framework to gain both latent means (brms) and SDs, following Taylor et al.’s (2023) approach. Although there were no major differences across the aggregation methods for the summary estimates, with all methods correlating highly with each other, there were nuanced differences. When comparing the traditional mean to the latent estimates, the variables of concreteness and imageability had a weakly non-linear relationship, a pattern not observed for the gender and valence variables. This may be explained by the distribution of the ratings along the scales, with both imageability and concreteness having a positive skew towards higher values on the scale, with no clearly defined neutral mid-point. This contrasts with the normal distributions observed for gender and valence, which also have a clearly interpretable neutral mid-point, suggesting that participant-level differences in the way participants use the scale are not accurately captured using a summary value such as the mean, but are captured in ordinal mixed-effects modeling approaches, as has been previously argued in the literature (see Liddell & Kruschke, 2018; Taylor et al., 2023; Veríssimo, 2021).

When investigating whether the different aggregation methods lead to differences when used to predict measures of lexical processing, we again note that there were only very subtle differences in the fixed-effect estimates, but no substantial differences in the overall inferences – all the variables were significant predictors for RTs and accuracy irrespective of the aggregation method used. Although some variables had estimates that appeared to differ slightly in magnitude, for example, imageability, we would highlight that larger coefficients in the model would not indicate that the aggregation method is any way superior. We would instead advocate again that the estimates derived from mixed models provide a more reliable estimate of the item's normative rating when compared to the traditional mean.

Given the computational demands when running the brms models (each model took approximately 14 h to complete using six chains and six cores on a standard computer device without a cluster), we would follow the recommendations of Taylor et al. (2023), who suggest that clmm models are a practical and efficient way to model data for rating norms, whilst also minimizing the influence of response bias that would be introduced from multiple participants, therefore offering a more suitable alternative to the traditional mean.

One benefit of using brms modeling can be observed if researchers are particularly interested in using estimates of item variance. For example, when inspecting the differences between the traditional estimates of variance in the ratings, specifically the SD, and comparing them to estimates derived from ordinal mixed-effects modeling, specifically the latent SD, we observed differences that aligned with Taylor et al.’s (2023) results. Response patterns appear to influence the distribution of SD values when compared to item estimates for the ratings, i.e., the mean or brms values, with lower SD values when the item estimates are at the edges of unidirectional scales, e.g., the lowest and highest values such as concreteness and imageability, or also at the neutral midpoint for gender and valence when the scale is more bipolar. This contrasts with the latent SD estimates, where the influence of response patterns appears to also be much more reduced, leading to estimates that more reliably capture variance in the item’s ratings.

Our final aim concerns the wider usage of the norms, specifically as they represent the largest multidimensional dataset of human-derived ratings for a large set of Czech words. Given the current interest and growth in datasets that can be used for research in psychology, linguistics, or the cognitive sciences, these norms will hopefully open up new avenues for researchers working on Czech, allowing them to address new research questions related to acquisition, processing, and use, or simply for controlling semantic variables for experimental design.

Moreover, these norms offer a valuable addition to the existing bank of megastudy resources already available for the dimensions of AoA, concreteness, gender, imageability, and valence, providing more opportunities for cross-linguistic research on semantic representations. Finally, the opportunity to utilize the raw and summary data alongside LLMs to extrapolate a larger dataset of norms that covers the wider lexicon would be a tangible future direction. Although work using LLMs for this purpose is still relatively novel, there remains a clear need for gold-standard datasets derived from human participants to benchmark such estimated values, with very little work published that has been applied to languages outside of heavily resourced languages.

In summary, we have presented in the present paper the largest multidimensional psycholinguistic norming dataset for Czech, providing not only the summary values across the dimensions for a large set of Czech words, but also the participant-level responses and a range of different aggregation summaries for both the item estimates of ratings and the variance within those ratings.

## Data Availability

The anonymized raw data and summary data, as well as the materials and stimuli from the experiments reported in this paper, can be accessed on the Open Science Framework at this link: https://osf.io/tqxfm and https://github.com/jamesbrandscience/czelex/.

## Footnotes

1 While there is no definitive criteria for how to classify whether or not a study is ‘large-scale’ (although see Keuleers & Balota, 2015), we did try to focus on published work that reports on at least 400 items for spoken languages and a slightly lower threshold for signed languages. It is certainly not a definitive list of all available data for each language.

2 We decided to set our lower threshold for the number of participants who knew the word to ten as this is in line with other rating studies, see, e.g., Lynott et al. (2020) and justifications presented by Motamedi et al. (2019) for iconicity.

## Code availability

All code used in the data preprocessing, filtering, and analysis from the experiments reported in this paper can be accessed on the Open Science Framework at this link: https://osf.io/tqxfm and https://github.com/jamesbrandscience/czelex/.
